# Epicardial Adipose Tissue and Psoriasis: A Systematic Review and Meta-Analysis

**DOI:** 10.3390/jcm13164761

**Published:** 2024-08-13

**Authors:** Xiaomei Chen, Hongmei Xiang, Jing Lu, Ming Yang

**Affiliations:** 1 Department of Dermatology, West China Hospital, Sichuan University, Chengdu 610041, China; chenmeir2008@126.com (X.C.); xhm@wchscu.cn (H.X.); 2 Medical Insurance Office, West China Hospital, Sichuan University, Chengdu 610041, China; 3 Chinese Cochrane Center, West China Hospital, Sichuan University, Chengdu 610041, China; 4 Center of Gerontology and Geriatrics, West China Hospital, Sichuan University, Chengdu 610041, China; 5 National Clinical Research Center for Geriatrics, West China Hospital, Sichuan University, Chengdu 610041, China

**Keywords:** epicardial adipose tissue, evidence-based medicine, psoriasis

## Abstract

**Background:** As a novel biomarker for cardiovascular diseases, epicardial adipose tissue (EAT) has been linked to psoriasis. We conducted an updated systematic review, building upon a previous report on the relationship between EAT and psoriasis. **Methods:** We searched Medline, Embase, and the Cochrane Central Register of Controlled Trials. The methodological quality of each study was assessed using the Newcastle–Ottawa Scale. The pooled mean difference (MD) or standardized mean difference (SMD) and the corresponding confidence interval (CIs) were calculated. **Results:** We included 10 studies with 1287 participants. Five of the included studies were of high methodological quality, while the other five were of moderate quality. The pooled data indicated that psoriasis patients had significantly increased EAT compared to individuals in the control group (SMD 1.53, 95% CI 0.61 to 2.45, 9 studies, 1195 participants). The subgroup analysis showed that psoriasis patients had significantly increased EAT thickness compared with the controls (SMD 2.45, 95% CI 0.73 to 4.17, 5 studies, 657 participants). Similarly, EAT area in single-slice CT images was significantly higher in the psoriasis group than in the control group (SMD 0.45, 95% CI 0.14 to 0.76, 2 studies, 195 participants). The EAT volume based on CT images appeared to be higher in the psoriasis group than in the control group, but the difference was not statistically significant (SMD 0.32, 95% CI −0.06 to 0.70, 2 studies, 343 participants). **Conclusions:** EAT, especially echocardiographic EAT thickness and CT-determined EAT area, was significantly associated with psoriasis, but CT-determined EAT volume was not.

## 1. Introduction

Psoriasis is a common, immune-mediated skin disease that affects up to 2% of the general population in Europe and North America [1]. In recent years, the understanding of psoriasis has evolved significantly. It is now recognized as a systemic inflammatory disease with far-reaching implications beyond the skin [1]. This systemic nature of psoriasis is evidenced by its association with multiple comorbidities, including arterial hypertension, dyslipidemia, diabetes mellitus, and mental health issues [2]. Recent data indicate that individuals with psoriatic disease are at substantially higher risk of developing cardiovascular diseases (CVDs) than those without the condition [3], while treating psoriasis may reduce the risk of CVDs by reducing inflammation [4].

The role of adipose tissues in inflammatory diseases has garnered increasing scientific interest, particularly in the context of psoriasis, a disease marked by both cutaneous manifestations and systemic inflammation [5]. Among various adipose depots, epicardial adipose tissue (EAT) is uniquely situated between the myocardium and visceral pericardium, positioning it at a crossroads of metabolic, cardiovascular, and inflammatory pathways [6]. Various adipose tissues, especially visceral fat, can produce inflammatory mediators contributing to systemic inflammation in psoriasis. However, EAT is uniquely positioned to have direct effects on cardiac and coronary structures due to its anatomical location. This direct interaction is hypothesized to play a significant role in the heightened cardiovascular risk associated with psoriasis [7].

Emerging research has highlighted EAT’s contribution to cardiovascular diseases, which are notably more prevalent in individuals with psoriasis [8]. The accumulation of EAT is strongly associated with poor prognosis in patients with heart failure [9] and can predict incident cardiovascular disease and mortality in patients with type 2 diabetes mellitus [10]. Therefore, EAT has been identified as an important risk factor for atherosclerosis and cardiovascular events and may be a promising new therapeutic target in CVDs [11].

As early as 2013, a cross-sectional study reported that increased EAT was a marker of CVD risk in psoriasis patients [12]. Subsequently, several observational studies have investigated the relationship between psoriasis and EAT [13,14,15,16]. In 2016, Wang et al. [17] performed a systematic review to summarize the findings from these studies [12,13,14,15,16]. Since then, some new studies have explored the association between EAT and psoriasis in different study populations, but with conflicting results [18,19,20,21]. We, therefore, conducted an updated systematic review to summarize all currently available evidence regarding the potential relationship between EAT and psoriasis.

## 2. Methods

This review adhered to the Preferred Reporting Items for Systematic Reviews and Meta-Analyses (PRISMA) guidelines [22].

### 2.1. Search

We searched the following databases via Ovid on 3 February 2024: (1) Ovid MEDLINE(R) and Epub Ahead of Print, In-Process, In-Data-Review & Other Non-Indexed Citations (from 1946); (2) Embase (from 1974); and (3) the Cochrane Central Register of Controlled Trials (January 2024). We used the terms “epicardial adipose tissue”, “epicardial fat tissue”, and “psoriasis” as either medical subject heading (MESH) terms or keywords. Table S1 shows the detailed search strategy. We also identified potentially relevant studies by screening all included publications’ references.

### 2.2. Inclusion and Exclusion Criteria

We applied the following inclusion criteria: (1) study population: individuals with psoriasis versus individuals without; (2) indicator: EAT measured by echocardiography, computed tomography (CT), or any other methods; (3) study type: case–control studies, cross-sectional studies, or cohort studies. We excluded reviews, case reports, meeting abstracts, editorials, corresponding letters, and non-English records.

### 2.3. Study Selection

To identify potentially relevant search records, two authors (XMC and HMX) independently screened the titles and abstracts. The full text of these search records was then independently reviewed by XMC and HMX in accordance with their inclusion and exclusion criteria. The reasons for exclusion were documented. When the two independent authors encountered divergent opinions regarding the eligibility of certain studies, the authors engaged in a structured dialogue, aiming to reach a unified decision through mutual agreement and careful consideration of each other’s viewpoints. In cases where a consensus could not be reached despite discussions, the arbitrator (MY) was consulted to reach an agreement.

### 2.4. Data Extraction and Management

A standard extraction template adapted from the Cochrane Good Practice Data Extraction Template [23] was used by one author (XMC) to extract essential data from the included studies. Data extraction forms were also checked by another author (MY). The original articles were rechecked to resolve discrepancies.

### 2.5. Methodological Quality Assessment

The methodological quality of each study was independently assessed by two authors (XMC and MY) using the Newcastle–Ottawa Scale (NOS) [24]. The NOS evaluates the quality of a study through three criteria: selection, comparability, and exposure. The maximum total score of NOS is nine points: four for quality of selection, two for comparability, and three for quality of exposures. The methodological quality of a study can be rated high (≥8 points), moderate (6 or 7 points), or low (≤5 points) [24]. When the two authors evaluating a study using the NOS had differing opinions on the quality assessment, their disagreements were resolved through discussion and consensus, including a detailed review of the criteria and the reasons for their differing scores. When the authors could not reach an agreement through discussion, they consulted the arbitrator (JL) whose role was to provide an objective evaluation based on the NOS criteria to resolve the dispute.

### 2.6. Data Synthesis

Wherever feasible, we conducted meta-analyses of the outcomes; otherwise, we provided a narrative description of the pertinent results. Considering that all outcomes were continuous data, we used mean difference (MD) when studies reported the outcome using the same scale and standardized mean difference (SMD) when studies reported the outcome using different scales. The pooled MD or SMD and the corresponding 95% confidence intervals (CIs) were determined by either a fixed-effects model or a random-effects model, depending on whether statistically significant heterogeneity is detected via the I2 test. I2 cutoffs have been established in 4 levels: 0%~40% for low heterogeneity; 30%~60% for moderate; 50%~90% for substantial, and 75%~100% for considerable heterogeneity [25].

Additionally, we performed subgroup analyses for different measurement methods used to detect EAT. Due to the small number of studies in each outcome, we did not perform meta-regression analyses or funnel plots. A two-sided *p*-value < 0.05 indicates statistical significance. We performed the data analyses using Review Manager (RevMan) Version 5.4.1.

## 3. Results

### 3.1. Results of Study Selection

**Figure 1:**
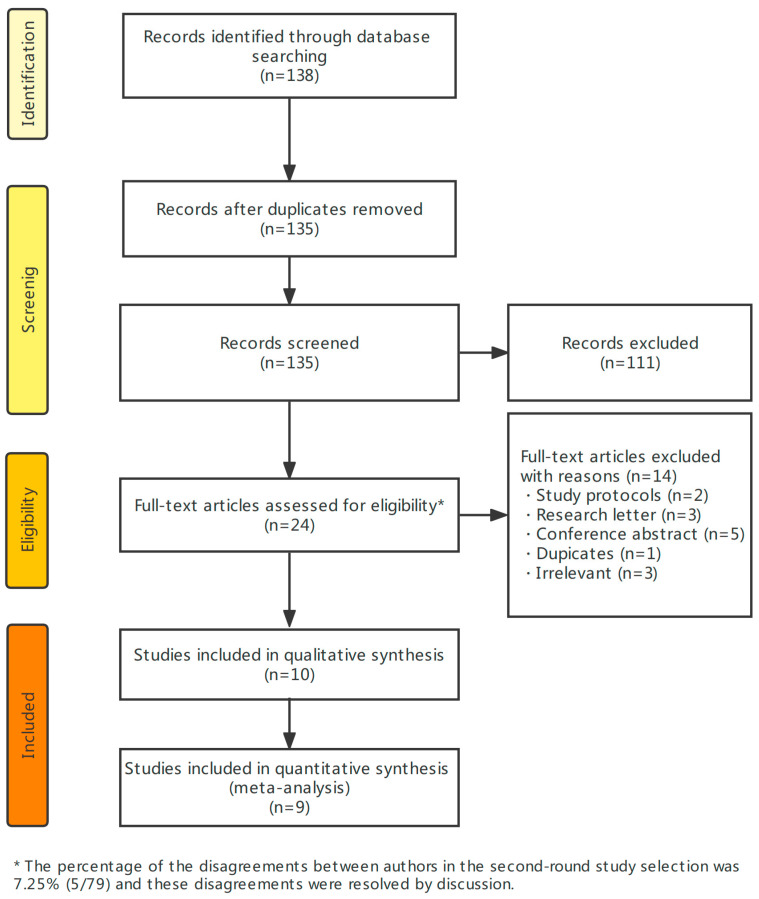
PRISMA flowchart for the study selection.

Figure 1 shows the PRISMA flowchart of the study selection. We initially identified 135 records after eliminating the duplicates. Of these, 111 records were discarded after screening the titles and abstracts, leaving 24 records for the full review. We excluded another 14 studies for various reasons (Figure 1) after reading the full texts. Thus, we finally included 10 studies [12,13,14,15,16,18,19,20,21,26] with a total of 1287 participants.

### 3.2. Characteristics of the Included Studies

Table 1 summarizes the characteristics of the included studies. All included studies were of case–control design. Six studies [12,13,14,15,18,19] were conducted in Turkey, the other four were conducted in India [20], Japan [21], Portugal [16], and the USA [26], respectively. Six studies [12,13,14,18,19,20] measured EAT thickness by echocardiography, two studies [15,21] measured EAT area by computed tomography (CT), and the other two studies [16,26] measured EAT volume by CT.

Table S2 shows the methodological quality of the included studies. Based on the NOS, five of the included studies [12,13,14,19,26] were graded as high quality, while the other five studies [15,16,18,20,21] were of moderate quality. 

### 3.3. EAT and Psoriasis

**Figure 2:**
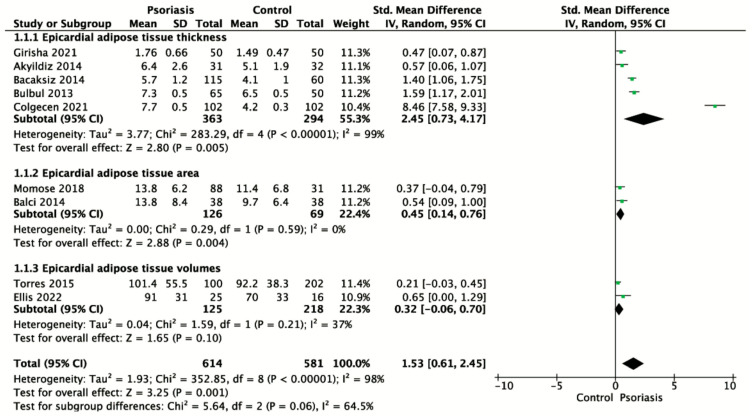
Forest plots of the standardized mean difference between the psoriasis patients and the controls for EAT stratified by the methods for EAT measurement. The diamond shape represents the pooled estimate from the meta-analysis, summarizing the combined results of all included studies. The green spot indicates a specific data point or an individual study’s effect size. CI, confidence interval; IV, inverse variance; SD, standard deviation [12,13,14,15,16,19,20,21,26].

We performed a meta-analysis including 9 studies [12,13,14,15,16,19,20,21,26] (614 psoriasis patients versus 581 control). The pooled data indicated that psoriasis patients had significantly increased EAT compared to individuals in the control group (SMD 1.53, 95% CI 0.61 to 2.45, 9 studies, 1,195 participants, I^2^ = 98%, *p* = 0.001, Figure 2). Additionally, one included study [18] could not be merged in the meta-analysis due to missing data. This study also showed that EAT was significantly higher in psoriatic patients than in the controls (3.1 mm vs. 2.8 mm, 1 study, 92 participants, *p* = 0.04).

We performed a subgroup analysis according to the different measurements of EAT (thickness, area, and volume). The pooled data showed that psoriasis patients had significantly increased EAT thickness compared with their counterparts in the control group (SMD 2.45, 95% CI 0.73 to 4.17, 5 studies, 657 participants, I^2^ = 99%, *p* = 0.005, Figure 2).

Similarly, EAT area in single-slice CT images was significantly higher in the psoriasis group than in the control group (SMD 0.45, 95% CI 0.14 to 0.76, 2 studies, 195 participants, I^2^ = 0%, *p* = 0.004, Figure 2). The EAT volume based on CT images appeared to be higher in the psoriasis group than in the control group, but the difference was not statistically significant (SMD 0.32, 95% CI −0.06 to 0.70, 2 studies, 343 participants, I^2^ = 37%, *p* = 0.10, Figure 2).

### 3.4. Other CVD Risk Factors and Psoriasis

**Figure 3:**
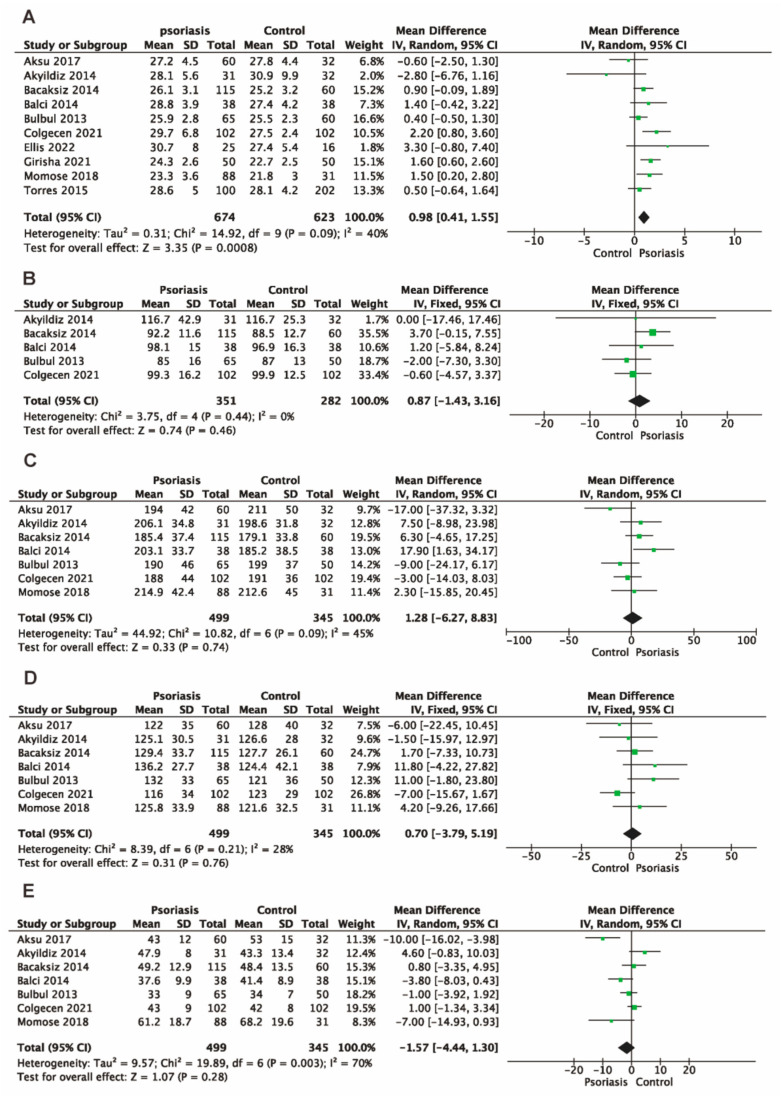
Forest plots for the mean difference between the psoriasis patients and the controls for body mass index (**A**), blood glucose (**B**), total cholesterol (**C**), low-density lipoprotein cholesterol (**D**), and high-density lipoprotein cholesterol (**E**). The diamond shape represents the pooled estimate from the meta-analysis, summarizing the combined results of all included studies. The green spot indicates a specific data point or an individual study’s effect size. CI, confidence interval; IV, inverse variance; SD, standard deviation [12,13,14,15,16,18,19,20,21,26].

Body mass index (BMI): the pooled data of a meta-analysis with 10 studies [12,13,14,15,16,18,19,20,21,26] indicated that patients with psoriasis showed a significantly higher BMI when compared to the control group (MD 0.98, 95% CI 0.41 to 1.55, 10 studies, 1297 participants, I^2^ = 40%, *p* = 0.0008, Figure 3A).

Blood glucose: Five studies [12,13,14,15,19] reported this outcome. The pooled data revealed no significant difference in blood glucose levels between the psoriasis group and the control group (MD 0.87, 95% CI −1.43 to 3.16, 5 studies, 633 participants, I^2^ = 0%, *p* = 0.46, Figure 3B).

**Figure 4:**
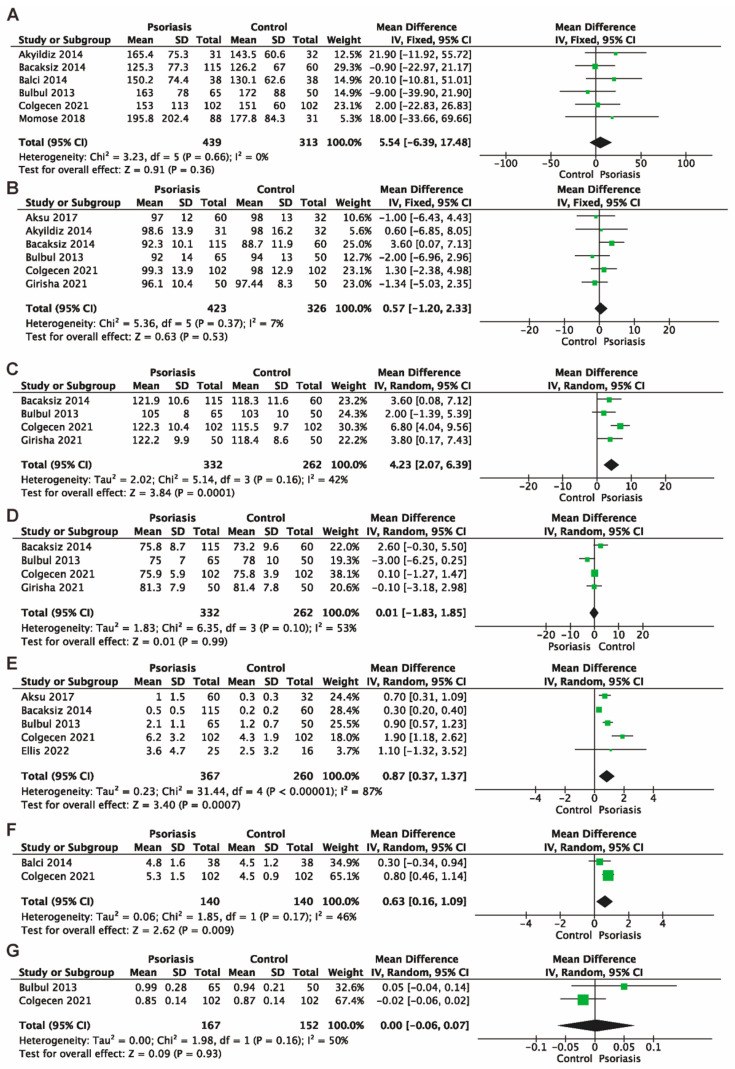
Forest plots for the mean difference between the psoriasis patients and the controls for serum triglyceride (**A**), waist circumference (**B**), systolic blood pressure (**C**), diastolic blood pressure (**D**), C-reactive protein (**E**), serum uric acid (**F**), and serum creatinine (**G**). The diamond shape represents the pooled estimate from the meta-analysis, summarizing the combined results of all included studies. The green spot indicates a specific data point or an individual study’s effect size. CI, confidence interval; IV, inverse variance; SD, standard deviation [12,13,14,15,16,18,19,20,21,26].

Serum lipids: Total cholesterol (TC), low-density lipoprotein (LDL), and high-density lipoprotein (HDL) were measured in seven studies [12,13,14,15,18,19,21], while plasma triglyceride (TG) was measured in six studies [12,13,14,15,19,21]. The meta-analyses found no significant difference between the psoriasis group and the control group concerning TC (MD 1.28, 95% CI −6.27 to 8.83, 7 studies, 844 participants, I^2^ = 45%, *p* = 0.74, Figure 3C), LDL (MD 0.70, 95% CI −3.79 to 5.19, 7 studies, 844 participants, I^2^ = 28%, *p* = 0.76, Figure 3D), HDL (MD −1.57, 95% CI −4.44 to 1.30, 7 studies, 844 participants, I^2^ = 70%, *p* = 0.28, Figure 3E), and TG (MD 5.54, 95% CI −6.39 to 17.48, 6 studies, 742 participants, I^2^ = 0%, *p* = 0.36, Figure 4A).

Waist circumference: the pooled data of a meta-analysis with 6 studies [12,13,14,18,19,20] found no significant difference between the psoriasis group and the control group (MD 0.57, 95% CI −1.20 to 2.33, 6 studies, 749 participants, I^2^ = 7%, *p* = 0.53, Figure 4B).

Blood pressure: Four studies [12,14,19,20] reported systolic blood pressure (SBP) and diastolic blood pressure (DBP). Compared with the control group, patients with psoriasis had elevated SBP (MD 4.23, 95% CI 2.07 to 6.39, 4 studies, 594 participants, I^2^ = 42%, *p* = 0.0001, Figure 4C). However, no significant difference was found in DBP between the 2 groups (MD 0.01, 95% CI −1.83 to 1.85, 4 studies, 594 participants, I^2^ = 53%, *p* = 0.99, Figure 4D).

C-reactive protein (CRP): Five studies [12,14,18,19,26] reported this outcome. The meta-analysis revealed that psoriasis patients had a significantly increased serum CRP level than the control group (MD 0.87, 95% CI 0.37 to 1.37, 5 studies, 627 participants, I^2^ = 87%, *p* = 0.0007, Figure 4E).

Uric acid: A meta-analysis of 2 studies [15,19] found that patients with psoriasis had a higher serum uric acid level than individuals in the control group (MD 0.63, 95% CI 0.16 to 1.09, 2 studies, 280 participants, I^2^ = 46%, *p* = 0.009, Figure 4F).

Creatinine: A meta-analysis of 2 studies [12,19] revealed no significant difference in serum creatinine between the psoriasis group and the control group (MD 0.00, 95% CI −0.06 to 0.07, 2 studies, 319 participants, I^2^ = 50%, *p* = 0.93, Figure 4G).

## 4. Discussion

Our review provided updated information regarding the association between EAT and psoriasis. The results indicated that EAT, especially EAT thickness measured by echocardiography and EAT area measured by CT images, was significantly higher in psoriasis patients than in healthy controls. However, the EAT volume measured by CT images was not significantly associated with psoriasis. Moreover, BMI, SBP, CRP, and uric acid were significantly associated with psoriasis, but waist circumference, blood glucose, serum lipids, DBP, and creatinine were not.

Psoriasis is not only a skin disease but also a systemic inflammatory condition linked to many extracutaneous comorbidities. In the past decade, there has been growing evidence that psoriasis patients are at a higher risk of multiple cardiovascular events including atherosclerosis, coronary artery disease, myocardial infarctions, and stroke [27]. Furthermore, observational studies have revealed a higher prevalence and incidence of common CVD risk factors, such as diabetes mullites, dyslipidemia, arterial hypertension, obesity, smoking, alcohol consumption, and CRP, in psoriasis patients [4,28]. Thus, screening psoriasis patients for cardiovascular risk factors may help in the primary prevention of CVDs in this high-risk population. Our finding that BMI, SBP, CRP, and uric acid were elevated in psoriasis patients is consistent with previous studies. However, these traditional CVD risk factors could not fully explain the increased risk of CVDs in psoriasis patients [29].

EAT, located between the myocardium and visceral pericardium, plays a crucial role in the proper functioning of the heart. Its proximity to the myocardium allows for direct interaction with coronary arteries, influencing cardiac metabolism [30]. EAT is not only a storage site for lipids but also an active endocrine organ that secretes various adipokines and cytokines, such as interleukin (IL)-1β, IL-6, TNF-α, resistin, and leptin [31]. These substances are pivotal in modulating cardiac function, particularly under stress conditions. The metabolic activity of EAT, including fatty acid uptake and release, contributes significantly to the energy supply of the myocardium, especially during increased cardiac workload [32]. Furthermore, EAT’s role in thermogenesis helps maintain optimal myocardial temperature, essential for efficient cardiac function [32].

The relationship between EAT and psoriasis comorbidities is an important area for consideration. Psoriasis is associated with numerous comorbidities, including cardiovascular diseases, metabolic syndrome, and non-alcoholic fatty liver disease. EAT may play a role in these associations. For example, increased EAT has been independently associated with coronary artery disease and atrial fibrillation [33]. EAT might serve as a link between cutaneous inflammation and cardiovascular risk. Additionally, EAT is metabolically active and may contribute to insulin resistance and dyslipidemia, both of which are common in psoriasis patients [1]. Understanding these relationships could provide a more comprehensive view of how EAT fits into the broader picture of psoriasis as a systemic inflammatory disease.

A previous meta-analysis has revealed the relationship between psoriasis and EAT with limited data [17]. Our review recruited and summarized the up-to-date evidence. We found a similar result that EAT was significantly associated with psoriasis. It is a logical assumption that if EAT thickness and area are related to psoriasis, EAT volume should also show a similar correlation. However, our systematic review interestingly found that while EAT thickness and area were correlated with psoriasis, EAT volume did not demonstrate a significant relationship. This lack of significant association between EAT volume and psoriasis, in contrast to the positive associations found with EAT thickness and area, is an intriguing finding that merits further exploration. Several factors might contribute to this discrepancy. (1) Measurement techniques: EAT volume is typically measured using CT or MRI, which provides a more comprehensive assessment of total EAT [34]. In contrast, EAT thickness is often measured by echocardiography at specific points, which may not fully represent the total EAT burden. (2) Sample size: our analysis of EAT volume included fewer studies compared to EAT thickness and area, which might have limited statistical power to detect a significant association. (3) Patient characteristics: The studies measuring EAT volume might have included patients with different disease severities or durations compared to those measuring thickness or area. Future research should aim to clarify these discrepancies by conducting larger studies that measure EAT using multiple techniques in the same patient cohorts.

Moreover, it is worth noting that the distribution of EAT may not be uniform. Studies have shown that EAT thickness can vary significantly depending on the measurement location. For example, Iacobellis et al. [34] found that EAT thickness over the right ventricle ranged from 1.9 to 15.7 mm. This non-uniform distribution could potentially affect the accuracy of EAT measurements and should be considered in future studies. Additionally, individual factors such as age, body mass index, and the presence of cardiovascular disease may influence EAT distribution, adding another layer of complexity to its assessment [34].

The exact cause of the increase in EAT among patients with psoriasis remains unknown. However, it is believed to be linked to chronic systemic inflammation associated with psoriasis, as well as the release of pro-inflammatory cytokines and adipocytokines from adipose tissue, such as Th17-related cytokines (IL-17, IL-6, and IL-8) [35]. Another potential cause of increased EAT in psoriasis may be a genetic predisposition. For example, there was evidence that IL-6 rs2069840 polymorphism presented in patients with psoriasis was associated with increased EAT volume [36]. Furthermore, lifestyle factors may contribute to the association between increased EAT and psoriasis. For example, smoking has been related to both increased EAT [37] and psoriasis [38], while aerobic exercise could significantly reduce EAT thickness [39]. Further research is needed to fully understand the exact cause of increased EAT in psoriasis patients.

Recent studies have provided new insights into the link between psoriasis and cardiovascular disease. For example, Romanelli et al. [40] reported tumor necrosis factor-α (TNF-α) in the myocardium and coronary arteries of psoriatic patients. This discovery is particularly significant as it provides direct evidence of the presence of inflammatory mediators in the cardiac tissue of psoriatic patients, even in younger individuals without a clinical history of heart disease. The presence of TNF-α in cardiac tissues of psoriatic patients could potentially be related to the inflammatory activity of EAT, providing a new perspective on the potential link between EAT, systemic inflammation, and cardiovascular risk in psoriasis.

The distribution and characteristics of EAT are complex and heterogeneous, making simplistic volume estimations based on area and thickness inadequate. The variability in EAT thickness, ranging widely from 1 mm to 25 mm, reflects differences in intra-abdominal fat accumulation across individuals, challenging uniform quantification methods [41]. Additionally, pathological conditions like coronary artery disease or diabetes mellitus significantly alter EAT’s properties, including its pro-atherogenic and pro-arrhythmogenic nature. These alterations show that EAT’s characteristics are dynamic and vary under different health scenarios [42].

There are several imaging modalities for measuring EAT. The thickness of EAT can be measured by CT, magnetic resonance imaging (MRI), or echocardiography, while the area or volume of EAT can be measured by CT and MRI. EAT volume measured by CT or MRI is the gold standard of EAT measurement, while EAT thickness and area are common surrogates for EAT volume [43]. Echocardiographic EAT thickness is usually measured from the echo-lucent area between the right ventricle and parietal pericardium on the parasternal short axis section [44]. CT-determined EAT area was usually calculated by tracing the contour of fat tissue defined by Hounsfield units (HU) from −190 to −30 on a single-slice CT image at the left main coronary artery origin level [45,46]. Notably, both echocardiographic EAT thickness and CT-determined EAT area may not accurately quantify EAT because EAT irregularly encircles the myocardium [12]. Moreover, although echocardiography is a convenient, feasible, cost-effective, and non-invasive method for measuring EAT, echocardiographic EAT thickness has been proven to be varied by devices and operators [47]. Our review indicated that EAT area and EAT thickness were significantly associated with psoriasis, whereas EAT volume was not. Therefore, further studies are needed to investigate the possible association between EAT and psoriasis in different clinical settings. Moreover, CT and MRI studies have highlighted variations in EAT’s density and fibrosis, indicating that a straightforward volumetric assessment based on area and thickness is insufficient [41]. However, none of the included studies addressed the density and fibrosis of EAT, which is warranted to be investigated in future studies.

Remarkably, the measurement of EAT volume needs CT or MRI, which need specific devices and software, high costs, and expertise [48]. CT also induces radiation exposure. These may limit their applications in clinical practice. However, chest CT has been commonly used for diagnosing many diseases in clinical practice or health check-ups [49]. The opportunistic use of these chest CT images provides an opportunity for measuring EAT volume. Furthermore, artificial intelligence (AI) algorithms are emerging to provide accurate and quicker methods to segment and quantify EAT, allowing for fast, robust, and fully automated quantification of EAT from non-enhanced CT images [50,51]. This technology may facilitate the study of EAT in large cohorts of psoriasis.

In this study, we used the NOS to evaluate the methodological quality of the included studies. The NOS is widely recognized as a valuable tool for assessing the quality of nonrandomized studies in meta-analyses, especially for observational studies [52]. Its strengths lie in its standardized approach to evaluating key quality parameters such as selection, comparability, and outcome across studies. This scale is appreciated for its straightforward application, making it a popular choice worldwide for such assessments [52]. However, there are limitations to the NOS that are important to consider. The scale can be somewhat subjective, particularly when it comes to fields with specific requirements or when evaluating cross-sectional studies [51]. Moreover, the NOS has been critiqued for its potential lack of detail in assessing certain study aspects, such as the exact method for ascertainment of exposure or the choice of appropriate outcomes of interest which might vary significantly between studies [53].

Our study has some limitations. First, we only included English literature. Thus, selection bias might be induced. Second, the clinical heterogeneity of the study populations across studies was significant. However, we were unable to perform subgroup analyses according to the types and severity of psoriasis due to missing the relevant data in the original studies. It would be interesting to explore EAT volume and the severity of psoriasis in future studies. Third, although we searched mainstream medical databases, we did not search some important databases, such as Web of Science and SCOPUS. Last, while our review focused on psoriasis and EAT, it is important to note that psoriatic arthritis, a common comorbidity of psoriasis, may also have implications for cardiovascular risk and potentially EAT. Future studies should investigate whether the presence of psoriatic arthritis influences the relationship between psoriasis, EAT, and cardiovascular outcomes.

## 5. Conclusions

Evidence from ten observational studies with small sample sizes and moderate-to-high methodological quality demonstrated that EAT, especially echocardiographic EAT thickness, and CT-determined EAT area, was significantly associated with psoriasis, but CT-determined EAT volume was not.

These findings suggest that EAT could potentially serve as a novel biomarker in psoriasis, highlighting several important directions for future research: (1) longitudinal studies to investigate whether changes in EAT correlate with psoriasis progression or response to treatment; (2) investigation into whether EAT measurements could serve as a predictor of cardiovascular risk in psoriasis patients, potentially informing clinical decision-making; (3) exploration of the molecular mechanisms linking psoriasis, systemic inflammation, and EAT accumulation; (4) evaluation of whether therapies targeting psoriasis-related inflammation also affect EAT and whether this correlates with cardiovascular outcomes; and (5) further studies to clarify the discrepancy between EAT volume and other EAT measurements in relation to psoriasis.

## Figures and Tables

**Table 1:** The characteristics of the included studies.

Study	Study Design	Country	Sample Size (m/w)		Mean Age, Years		Measurement Methods	EAT	
			Psoriasis	Control	Psoriasis	Control		Psoriasis	Control
Colgecen, 2021 [19]	Case–control	Turkey	46/56	46/56	40.9 ± 8.6	43.1 ± 6.7	Echo	7.7 ± 0.5 mm	4.2 ± 0.3 mm
Girisha, 2021 [20]	Case–control	India	50 *	50 *	41.8 ± 12.2	42.2 ± 12.3	Echo	1.76 ± 0.66 mm	1.49 ± 0.47 mm
Aksu, 2017 [18]	Case–control	Turkey	37/23	16/16	44 ± 14	42 ± 8	Echo	3.1 (2.4–3.5) mm	2.8 (2.0–3.2) mm
Akyildiz, 2014 [13]	Case–control	Turkey	14/17	13/19	42 ± 11.1	41.1 ± 6.8	Echo	6.4 ± 2.6 mm	5.1 ± 1.9 mm
Bacaksiz, 2014 [14]	Case–control	Turkey	62/53	28/32	33.6 ± 6.0	32.5 ± 8.3	Echo	5.7 ± 1.2 mm	4.1 ± 1.0 mm
Bulbul, 2013 [12]	Case–control	Turkey	39/26	28/22	41.1 ± 3.3	40.5 ± 3.8	Echo	7.3 ± 0.5 mm	6.5 ± 0.5 mm
Momose, 2018 [21]	Case–control	Japan	62/26	13/18	57.2 ± 14.8	58.7 ± 14.5	CT	13.8 ± 6.2 cm^2^	11.4 ± 6.8 cm^2^
Balci, 2014 [15]	Case–control	Turkey	26/12	26/12	42.2 ± 15.0	39.8 ± 13.5	CT	13.8 ± 8.4 cm^2^	9.7 ± 6.4 cm^2^
Ellis, 2022 [26]	Case–control	USA	14/11	5/11	46 ± 6	47 ± 7	CT	91 ± 31 mm^3^	70 ± 33 mm^3^
Torres, 2015 [16]	Case–control	Portugal	64/36	130/72	47.4 ± 10.8	54.4 ± 10.1	CT	101.4 ± 55.52 mm^3^	92.2 ± 38.33 mm^3^

Continuous variables were presented as mean ± standard deviation (SD) or median with quartile range. * The sex of the participants was not reported in this study. CT, computed tomography; EAT, epicardial adipose tissue; Echo, echocardiography; m/w, men/women.

## Supplementary Materials

The following supporting information can be downloaded at: https://www.mdpi.com/article/10.3390/jcm13164761/s1, Table S1: The search strategy for Medline, Embase, and Cochrane Central Register of Controlled Trials via OvidSP; Table S2: The methodological quality of the included studies based on the Newcastle–Ottawa Scale.
