# Supplementary material for: Epicardial Adipose Tissue and Psoriasis: A Systematic Review and Meta-Analysis
Source: J Clin Med. 2024 Aug 13;13(16):4761. doi: 10.3390/jcm13164761 (PMC11355870; doi:10.3390/jcm13164761)
Supplement: Supplementary file 1 [file jcm-13-04761-s001.zip › jcm-3054200-supplementary.pdf]

Table S1. The search strategy for Medline, Embase, and Cochrane Central Register of Controlled Trials via OvidSP

1 exp epicardial adipose tissue/
2 exp epicardial fat tissue/
3 exp psoriasis/
4 psoria$. mp.
5 3 or 4
6 ((pericardi$ or epicardia$ or paracardi$) and (fat$ or adipos$)). mp.
7 1 or 2 or 6
8 5 and 7

Table S2. The methodological quality of the included studies based on the Newcastle-Ottawa Scale

Study	Selection	Comparability	Exposure	Total score
Ellis 2022	3	4	3	8
Colgecen 2021	3	2	3	8
Girisha 2021	2	2	2	6
Momose 2018	2	2	2	6
Aksu 2017	2	2	3	7
Torres 2015	3	2	2	7
Akyildiz 2014	3	2	3	8
Bacaksiz 2014	3	2	3	8
Balci 2014	2	2	3	7
Bulbul 2013	3	2	3	8
